# Neurological vertigo in the emergency room in pediatric and adult age: systematic literature review and proposal for a diagnostic algorithm

**DOI:** 10.1186/s13052-022-01313-7

**Published:** 2022-07-27

**Authors:** Noemi Pellegrino, Vincenzo Di Stefano, Eleonora Rotondo, Alessandro Graziosi, Marianna Gabriella Rispoli, Angelo Torrente, Antonino Lupica, Filippo Brighina, Umberto Raucci, Pasquale Parisi

**Affiliations:** 1 Neonatal Intensive Care Unit, Pescara Public Hospital, Pescara, Italy; 2 Department of Biomedicine, Neuroscience and Advanced Diagnostic (BIND), University of Palermo, Palermo, Italy; 3 Department of Pediatric and Neonatology, Ciriè Hospital, Ciriè, Piemonte, Italy; 4 Department of Primary Care, ASL Viterbo, Viterbo, Italy; 5 Department of Neuroscience Imaging and Clinical Sciences, University G. D’Annunzio, Chieti, Italy; 6 Department of Emergency, Acceptance and General Pediatrics, Bambino Gesù Children’s Hospital, IRCCS, Rome, Italy; 7 Chair of Pediatrics, NESMOS Department, Faculty of Medicine & Psychology, “Sapienza” University, Sant’Andrea Hospital, Rome, Italy

**Keywords:** Vertigo, Adulthood, Childhood, Emergency department

## Abstract

Neurological vertigo is a common symptom in children and adults presenting to the emergency department (ED) and its evaluation may be challenging, requiring often the intervention of different medical specialties. When vertigo is associated with other specific symptoms or signs, a differential diagnosis may be easier. Conversely, if the patient exhibits isolated vertigo, the diagnostic approach becomes complex and only through a detailed history, a complete physical examination and specific tests the clinician can reach the correct diagnosis. Approach to vertigo in ED is considerably different in children and adults due to the differences in incidence and prevalence of the various causes. The aim of this systematic review is to describe the etiopathologies of neurological vertigo in childhood and adulthood, highlighting the characteristics and the investigations that may lead clinicians to a proper diagnosis. Finally, this review aims to develop an algorithm that could represent a valid diagnostic support for emergency physicians in approaching patients with isolated vertigo, both in pediatric and adult age.

## Introduction

Vertigo can be defined as a disorder of space sensitivity classically described as an unpleasant illusion of motion of the patient or the environment [1–3]. It is an acute and severe symptom that may affect quality of life provoking significant apprehension along with significant occupational impacts. It is a common reason for ED presentation and can be isolated or associated with other symptoms. Conversely, dizziness is a different sensation that can be described as an altered spatial perception without a false sense of motion [3]. Vertigo and dizziness might often be confused by patients and clinician; however the former often refers to an objective sense of external motion, while the latter refers to a subjective sense of instability.

Although vertigo is usually a symptom of peripheral causes as opposed to dizziness, neurological conditions may often presented with vertigo and the spectrum of differential diagnoses is broad and different for adult and pediatric population. The main causes of adulthood neurological vertigo, primarily represented by acute vascular injuries, are uncommon or totally absent in childhood [4, 5]. Moreover several peculiar etiologies of the pediatric neurological vertigo, as opposed to adults, are characterized by favorable prognosis [6]. Hence, the diagnosis is challenging, especially when the vertigo is the only clinical sign at the onset of the symptomatology [7].

A proper diagnostic evaluation essentially includes a stepwise detailed history, a careful physical examination and further tests based on clinical indications [4]. A meticulous medical history taking is the first step to distinguish the peripheral causes of vertigo from the central ones. Assessing intensity, timing and triggers of vertigo may be helpful, even if this approach shows major limitations [8]. For example, patients with acute, spontaneous, isolated vertigo may suffer either from acute vestibular neuritis/labyrinthitis or cerebellar stroke. Conversely, patients with spontaneous, episodic vertigo may be affected by vestibular migraine (VM) but also suffer from recurrent, stereotyped transient ischemic attacks [9]. In adults positional vertigo, mainly attributed to benign paroxysmal positional vertigo (BPPV), may also have a central origin, especially when persistent and associated with nystagmus [9]. Another aspect to be investigated is the presence of concurrent symptoms; however, they are often misleading or missing initially (e.g. headache in VM, hypoacusis in anterior inferior cerebellar artery (AICA) strokes) [8]. In terms of physical examination it is mandatory to assess the presence of nystagmus which may be horizontal, exhaustible, inhibited by fixation and worsened by head shaking in the peripheral forms, while it is commonly vertical or rotatory in the central ones [10]. The causative origin of vertigo may also be evaluated using diagnostics indexes that combine both history and patients’ clinical characteristics (e.g., ABCD^2^, CATCH^2^, STANDING scores) [8]. Lastly, advanced examinations may be crucial, such as the association of normal vestibulo-ocular reflex (VOR) with head impulse testing (HIT), gaze-evoked nystagmus and the presence of skew deviation at the test of skew (HINTS) that suggests a central cause of vertigo [11].

To date few studies have tried to validate specific diagnostic approaches for neurological vertigo and, in particular for patients with vertigo as exclusive clinical manifestation, there are no standardized diagnostic algorithms [4]. The aim of this systematic review is to identify and describe the characteristics of the neurological disorders presenting, at the onset, with isolated vertigo in childhood and adulthood and to propose a diagnostic algorithm to help clinicians working in ED.

## Methods

**Fig. 1:**
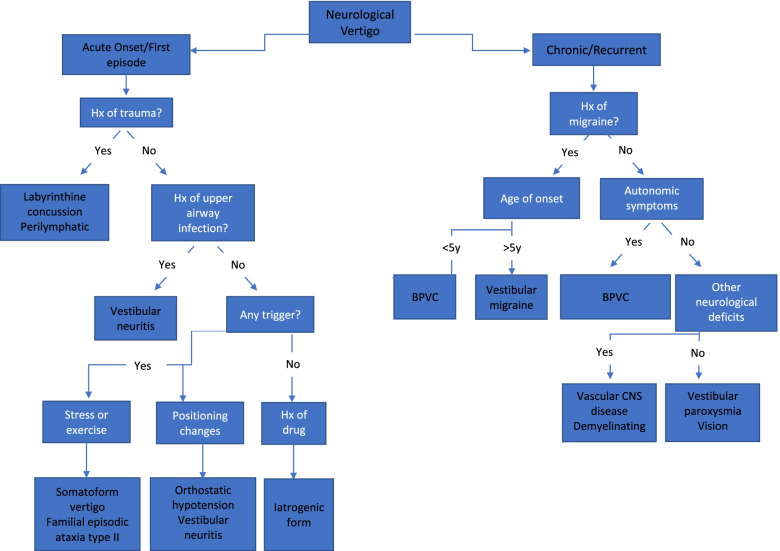
Algorithm for evaluation of neurological vertigo in children

**Fig. 2:**
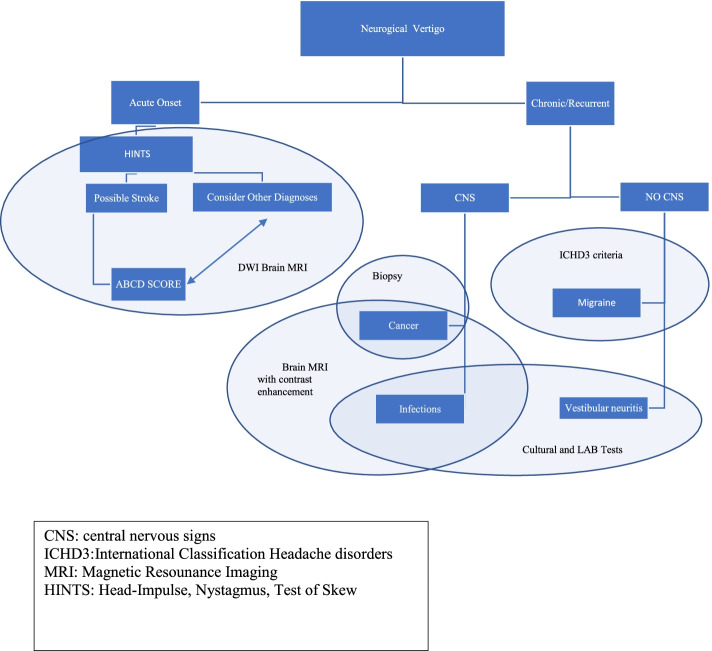
Algorithm for evaluation of neurological vertigo in adults

We carried out a systematic review of the literature using PubMed/NCBI database, through a comprehensive MEDLINE search, in order to identify all the available studies describing the isolated neurological vertigo. The following search words were used: “isolated neurological vertigo” and “isolated vertigo children”. The search was conducted between May 19th, 2020 and July 10th, 2020 and it collected studies published between March 1989 and July 2020. We included all original articles (case reports, case series, prospective and retrospective observational studies) written in English, in which subjects presented with isolated vertigo. Our research included not only diseases characterized by vertigo as the only clinical manifestation, but also all the potential causes that, even if usually associated with other symptoms, may initially present with vertigo as exclusive complaint or could be associated to signs highlighted only during the clinical examination. We included studies involving both pediatric and adult population. All the reviewers worked independently in selecting the studies and extracting information about epidemiological, clinical and diagnostic features of patients presenting with isolated vertigo. Finally, we proposed two distinct diagnostic algorithms to ascertain the differential diagnosis of isolated neurological vertigo in pediatric (Fig. 1) and adult (Fig. 2) patients, using key elements of the history and physical examination.

## Results

**Fig. 3:**
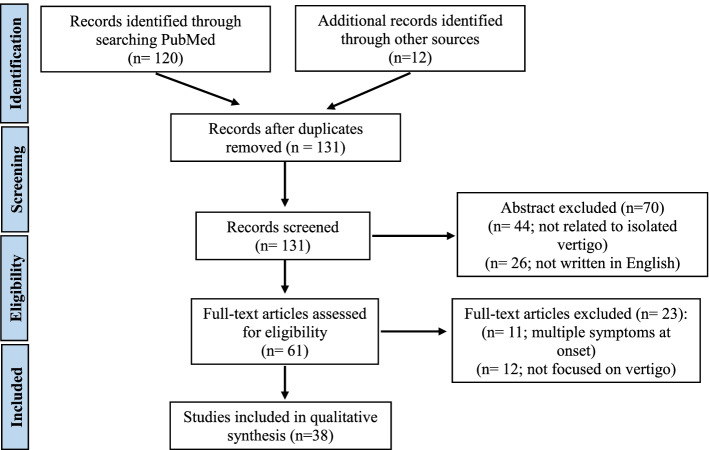
PRISMA diagram

Our research yielded overall 120 abstracts and a further 12 papers were later added following an additional screening of references from unselected papers. After checking for duplicates we excluded 70 records by reviewing articles’ abstracts. After a detailed examination of the full texts of the 61 remaining articles, we found 38 papers meeting our inclusion/exclusion criteria, which were subsequently included in the qualitative synthesis, 12 related to pediatric patients and 26 related to adult ones (Fig. 3, Tables 1 and 2). We found that isolated vertigo is associated with several adult and pediatric diseases, that are listed in Tables 3 and 4.

**Table 1:** Characteristics of the selected pediatric studies

Study, country	Age range	Study design	Time frame	No. of subjects	Comments
Lanzi G. et al., 1994 [12] Italy	12.5 to 24.2 years. old	Prospective study	Not specified	47	Vertigo is a rare symptom in patients with juvenile migraine
Salman MS. et al., 2017 [13] Canada	6 to 32.75 years old	Retrospective study	1991 to 2008	185	Vertigo/dizziness are often associated with chronic ataxia. Genetic, metabolic and inflammatory disorders should be considered in these patients
Langhagen T. et al., 2013 [14] Germany	1.4 to 18 years old	Retrospective study	November 2009 to April 2012	168	Migraine-related vertigo is the most common cause of vertigo and dizziness in children and adolescents
Ramantani G. et al., 2009 [15] Germany	9 years old	Case report	Not specified	1	Episodes of vertigo can be rarely associated with tuberous sclerosis
Caldarelli M. et al., 2007 [16] Italy	2 months to 16 years	Retrospective study	January 1993 to August 2005	30	Vertigo is a complaint presenting in 30% of pediatric patients with symptomatic Chiari malformation Type I
Kalashnikova LA. et al., 2005 [17] Russia	7 to 72 years	Retrospective study	Not specified	25	A sudden onset of vertigo can be a clinical manifestation of cerebellar infarcts
Bucci MP. et al., 2004 [18] France	6 to 15 years old	Prospective study	Not specified	12	Vertigo in children with normal vestibular function can be associated with abnormal vergence latency
Russell G. et al., 2009 [19] Scotland	School age	Epidemiological study	Not specified	2165	Paroxysmal vertigo is common in childhood and it appears to cause few major problems to the affected children
D’Agostino R. et al., 1997 [1] Italy	4 to 14 years old	Retrospective study	1985 to 1989	282	Vertigo as isolated symptom is the most frequent clinical presentation in childhood. Paroxysmal benign vertigo is the second most frequent cause of vertigo
Raucci U. et al., 2015 [7] Italy	3 to 18 years old	Retrospective study	January 2009 to December 2013	616	Vertigo is most frequently related to benign conditions such as migraine and syncope. Early recognition of associated signs or symptoms is mandatory to identify need for further investigations
Lehnen N. et al., 2015 [20] Germany	8 to 12 years old	Case report	10-year period	3	Vestibular paroxysmia should be considered in children with short, frequent vertiginous episodes
Mugundhan K. et al., 2011 [21] India	13 to 65 years old	Case report	Not specified	5	The presence of recurrent episodes of vertigo is typical in familial episodic ataxia type II. Cerebellar function tests can be completely normal between the attacks

**Table 2:** Characteristics of the selected adult studies

Study, country	Age range	Study design	Time frame	No. of subjects	Comments
Joshi P. et al., 2020 [22] New Zealand	28 to 65 years old	Case series	Not specified	7	BPPV is the most common cause of positional vertigo
Grad A. et al., 1989 [23] USA	40 to 81 years old	Retrospective study	1974 to 1987	84	The sudden onset of vertigo lasting minutes in a patient with known cerebrovascular disease strongly suggests an ischemic cause
Norrving B. et al., 1995 [24] Sweden	50 to 75 years old	Prospective study	Not specified	24	A caudal cerebellar infarction may easily be misdiagnosed as a labyrinthine disorder, and it is found to be the cause in one in fourpatients presenting with isolated acute vertigo
Kim GW. et al., 1996 [25] Korea	Not specified	Prospective study	August 1994 to February 1995	152	Vertigo as a manifestation of stroke may not be an infrequent symptom
Casani AP. et al., 2013 [26] Italy	47 to 80 years old	Retrospective study	2007 to 2011	11	Pseudo-acute peripheral vertigo is not an uncommon diagnosis in otoneurological practice
Doijiri R. et al., 2016 [27] Japan	56 to 79 years old	Retrospective study	2005 to 2015	221	In this study stroke was found in 11% of patients with isolated vertigo or dizziness attack. The posterior inferior cerebellar artery area is frequently implicated for isolated vertigo or dizziness
Hesselbrock RR 2017 USA	40 to 42 years old	Case reports	Not specified	2	Accurate assessment of patients with acute vestibular symptoms can be challenging, Central causes of isolated acute vestibular symptoms are uncommon
Perloff MD. et al., 2017 [28] USA	Mean age 59.8 ± 16.7	Retrospective study	January 2005 to January 2010	136	There is an important proportion of cerebellar stroke among emergency department in patients with isolated dizziness
Wang Y. et al., 2018 China	Mean age 58.5 ± 12.3 for central vertigo and 52.1 ± 8.8 for peripheral vertigo	Retrospective study	January 2014 to July 2016	87	Patients with isolated vertigo and three or more risk factors are at higher risk for central vertigo
Lee H. et al., 2009 [29] Korea	23 to 93 years old	Prospective study	January 2000 to July 2008	82	Labyrinthine dysfunction of a vascular cause usually leads to combined loss of both auditory and vestibular functions
Paul NL. et al., 2012 UK	Mean age 75.9 ± 11.8 for carotid stroke and 73.3 ± 13.1 for vertebrobasilar stroke	Prospective study	April 2002 to March 2010	1141	In patients with vertebrobasilar stroke, preceding transient isolated brainstem symptoms are common but rarely satisfy traditional definition of TIA
Lee SU. et al., 2015 [30] Korea	33 to 73 years old	Retrospective study	2003 to 2014	18	Presence of central vestibular signs allows bedside differentiation of isolated vestibular syndrome
Parthasarathy R. et al., 2016 [31] Canada	76 years old	Case report	Not specified	1	Hypoperfusion to the flocculonodular lobe supplied by the anterior inferior cerebellar artery is likely a cause for intermittent vertigo
Lee H. et al., 2002 [32] Korea	17 to 74 years old	Prospective study	March 2000 to July 2001	72	Migraine should be considered in the differential diagnosis of isolated recurrent vertigo of unknown cause
Kim DD. et al., 2019 [33] Canada	60 s years old	Case report	2019	1	Chronic naturopathic over- the-counter products intake may cause a subacute progressive cerebellar syndrome manifesting also with vertigo
Adzic-Vukicevic T. et al., 2019 [34] Serbia	66 years old	Case report	2019	1	Cryptococcosis may present even in immunocompetent patients and may show central nervous system involvement with vertigo
Pula JH. et al., 2013 [35] USA	19 to 55 years old	Prospective observational study	1999 to 2011	7	Multiple sclerosis is an uncommon cause of acute vestibular syndrome
Kremer L. et al., 2014 [36] France, USA, UK, Japan, Canada, Germany	Mean age 44.2	Prospective observational study	Not specified	258	Brainstem involvement occurs in about one-third of patients with NMO and NMOSD; vertigo or vestibular ataxia occur in 1.7% of patients
Lee JY. et al., 2019 [37], Korea	20 to 80 years old	Retrospective analysis	January 2012 to January 2015	133	Vestibular neuritis is characterized of rotational vertigo that last for over a day but the clinical course and the characteristics depends on the involvement site of the nerve
Roberts RA., 2018 [38] USA	60 years old	Case report	Not specified	1	Patients using biologic disease-modifying antirheumatic drugs could be at an increased risk for recurrent vestibular neuritis, with possible viral pathogenesis
Unal M. et al., 2006 [39]	51 years old	Case report	Not specified	1	It is important to consider Arnold-Chiari type I malformation in the differential diagnosis of adult vertigo cases
Spacey S.et al., 2003	2 to 32 years old	Review	Not specified	Not specified	Episodic ataxia type 2 (EA2) is characterized by paroxysmal attacks of ataxia, vertigo, and nausea. Onset is typically in childhood or early adolescence
Rispoli MG. et al., 2019 [40]	71 years old	Case report	March 2015	1	New missense mutation in the ATP1A2 gene is associated with atypical sporadic hemiplegic migraine, a disease possibly manifesting with vertigo
Di Stefano V. et al., 2020 [41]	71 years old	Case report	Not specified	1	A rare case of atypical BHS due to compression of non-dominant vertebral artery with anatomical variants, resulting in stereotyped and reversible PICA syndrome
Potter BJ. et al., 2014 [42]	90 years old	Case report	Not specified	1	A subclavian steal syndrome may occur when a significant stenosis in the subclavian artery compromises distal perfusion to the internal mammary artery, vertebral artery, or axillary artery
Jiang Y. et al., 2020 [43]	34 years old	Case report	Not specified	1	Frontal lobe epilepsy is a common neurological disorder with a broad spectrum of symptoms; it rarely presents with vertigo

**Table 3:** Differential diagnosis of isolated neurological vertigo in childhood

Differential Diagnosis	Incidence/Prevalence	Main Features	Clues for Differential	Examination Required	References
**Cephalalgia**					
Vestibular migraine	24% Mainly > 5yo	Vestibular symptoms (rotator vertigo) temporarily with migraine Time: 5 min or 72 h	Episodic vertigo, age > 5yo, attacks lasting minutes to hours, association at least in some cases with migraine headache or migrainous phenomena	Physical exam and vestibular tests	Lanzi et al. 1994 [12], D’Agostino et al. 1997 [1], Russell et al., 1999 Langhagen T et al. 2013 [14], Raucci et al. 2015 [7]
Benign Paroxysmal vertigo of childhood	14 to 18% Mainly < 5yo F > M	Episodic syndrome with short, non-epileptic, recurrent attacks of subjective or objective vertigo, which resolve spontaneously	Episodic vertigo, age < 5yo, attacks lasting seconds to minutes (to hours) without migraine headache	Clinical exam and instrumental investigations (absence of hearing impairment)	D’Agostino et al. 1997 [1], Russell et al., 1999 [19] Langhagen T et al. 2013 [14], Raucci et al. 2015 [7]
**Brain tumour and/or malformation**					
Expansive endocranial pathologies and/or malformation	Rare	Vertigo, neurological symptoms, haedache	Association with additional neurologic deficits but neuroimaging is essential	Clinical exam and neuroimaging	D’Agostino et al., 1997 [1] Caldarelli M. et al., 2007 [16] Raucci et al., 2015 [7]
**Vascular diseases**					
Neurovascular diseases	Rare	Vertigo, neurological symptoms, sincope	Association with additional neurologic deficits but neuroimaging is essential	Clinical exam and neuroimaging	Kalashnikova et al.,2005 [17] Raucci et al., 2015 [7]
**Demyelinating diseases**					
Demyelinating diseases	Rare	Vertigo, multidirectional nystagmus	Association with additional neurologic deficits but neuroimaging is essential	Vestibular tests, MRI	D’Agostino et al. 1997 [1], Raucci et al. 2015 [7], Salman M. et al., 2017 [13]
**Inflammatory disease**					
Vestibular neuritis	16% Mainly > 5yo and adolescents	Sudden onset of severe vertigo, sometimes associated with nausea and vomiting	Vertigo can be intensified by small changes in head position	Electronystagmography, thermal caloric testing	D’Agostino et al., 1997 [1] Raucci et al., 2015 [7]
**Others**					
Somatoform vertigo	2.5 to 16% Mainly adolescent girls	Vertigo organically not sufficiently explained	Normal findings on physical exam and diagnostic evaluation	Psychiatric consultation	D’Agostino et al., 1997 [1] Raucci et al., 2015 [7]
Head and/or cervical trauma	7–10% of pediatric giddiness	Isolated vertigo or vertigo associated with hearing loss or others symptoms	History of previous trauma	Imaging of head/cervical chord	Raucci et al., 2015 [7]
Orthostatic hypotension	3–9% of pediatric giddiness	Isolated vertigo or associated with autonomic symptoms, including syncope	Sudden drop in blood pressure after change in positioning	Blood pressure measurement, tilt test	Raucci et al., 2015 [7]
Vestibular paroxysmia	4% of pediatric giddiness	Frequent episodes of vertigo, several times in a day, lasting for seconds to minutes, regardless of posture	Good response to carbamazepine or oxcarbazepine	Neuroimaging	Lehnen N et al., 2015 [20]
Iatrogenic form	Rare	Rarely cause of isolated vertigo	History of drug use or abuse	None/Urine analysis/toxicology screening	D’Agostino et al., 1997 [1]
Tuberous Sclerosis	Only report	Only one case described child with episodes of vertigo and headache	Presence of amartomas	Cranial MRI/abdomen ultrasound	Ramantani. et al. 2009 [15]
Familial episodic ataxia type II	Rare	Stress or exercise-induced vertigo and ataxia	Carbonic anhydrase inhibitor, such as acetazolamide, produces a complete response to vertigo	Brain MRI	K. Mugundhan, 2011 [21]
Anisometropia and other ocular abnormalities	Rare	Sensory mismatch	Resolution with ophthalmological treatment	Ophthalmological examination	Bucci M.P. et al., 2004 [18]

**Table 4:** Differential diagnosis of isolated neurological vertigo in adulthood

Differential Diagnosis	Incidence/Prevalence	Main Features	Clues for Differential	Examination Required	References
**Primary or secondary brain tumours**					
Cerebellar lymphoma	CNS lymphoma represents 2–6% of all primary brain neoplasms (1.34 cases per million people); cerebellar involvement presents in only 9% of cases	Sudden onset of vertigo associated with vomiting	Neurotological evaluation: atypical nystagmus patterns during diagnostic maneuvers may raise suspicion of central pathology	Brain MRI with contrast enhancement and biopsy	Joshi et al., 2020 [22]
Cerebellar metastases	98,000–170,000 cases of brain metastases/year; metastases to the cerebellum accounts for 10–15% of all brain metastasis	Onset with severe headache, associated with nausea and vomiting, followed by positional vertigo and unsteady standing	Neurotological evaluation: atypical nystagmus patterns during diagnostic maneuvers may raise suspicion of central pathology	Brain MRI with contrast enhancement	Joshi et al., 2020 [22]
Infratentorial gliomas	Incidence of glioma is about 6.0 per 100,000 person-years; infratentorial gliomas represent 4.6% of all gliomas	Occasional attacks of vertigo and nausea lasting less than 30 seconds, related to changes in head position	Neurotological evaluation: atypical nystagmus patterns during diagnostic maneuvers may raise suspicion of central pathology	Brain MRI with contrast enhancement	Joshi et al., 2020 [22]
**Ischemic stroke**					
Cerebellar stroke	2–3% of 600,000 stroke-year in the United States. Presumed stroke etiologies: atherosclerotic occlusive lesions of the vertebral artery (32%), in situ branch artery disease (25%), cardioembolism (10%), vertebral artery dissection (5%)	Sudden onset of rotational vertigo associated with neurovegetative symptoms (nausea and vomiting). Sometimes concomitant headache or unilateral hearing loss	Head Impulse Test (HIT) is positive in acute peripheral vertigo (APV) and negative in cerebellar strokes (pseudo-APV). Delayed onset of other central symptoms/signs is not uncommon	CT scan, MRI and neurotologic examination	Grad A et al. 1989 [23], Norrving et al. 1995 [24]; Kim GW et al. 1996 [25], Casani et al., 2013 [26], Joshi et al., 2020 [22], Doijiri et al., 2016 [27], Hesselbrock, 2017; Perloff et al., 2017 [28], Wang et al., 2018;
Pons stroke	7% of all ischemic strokes, 15–20% of posterior circulation ischemia. One in ten non-traumatic intracerebral hemorrhages is located in the pons	Vertigo and vomiting, falls and pointing towards the affected side, direction fixed nystagmus towards the unaffected side	Impairment of smooth pursuit eye movements may be present	MRI and neurotologic examination	Norrving et al. 1995 [24]; Kim GW et al. 1996 [25], Lee et al. 2009; Doijiri et al., 2016 [27], Wang et al., 2018
Medulla oblongata stroke	Not found exact incidence/prevalence. In a study: annual incidence of posterior circulation infarction is 18 per 100 000 person years in an Australian study (Dewey et al. 2003) 10–20% of them may cause acute vestibular syndrome	Diverse patterns of spontaneous nystagmus, gaze-evoked nystagmus and head-shaking nystagmus, possible otolithic dysfunction, subjective visual vertical (SVV) tilt, presence of at least one component of the ocular tilt reaction (OTR)	Less than a third of patients have abnormal ocular and cervical vestibular-evoked myogenic potentials (VEMPs) in lateral medullary infarction. Abnormal VEMPs are seen in about one-half of patients in medial medullary infarction	MRI and neurotologic examination	Paul et al., 2013; Sun-Uk Lee et al., 2015; Doijiri et al., 2016 [27], Wang et al., 2018
Persistent trigeminal artery (PTA)	Prevalence 0.1%-0.2% of cerebral angiograms	Isolated intermittent vertigo, followed by anterior and posterior circulation ischemic strokes symptoms	CT angiography evidence of PTA and CT signs of ischemic stroke	CT angiography	Parthasarathy, et al. 2016 [31]
**Cephalalgia**					
Migraine	*The prevalence of migraine according to IHS criteria was higher in the isolated recurrent vertigo group (61.1%) than in the control group (10%; p < 0.01)	isolated recurrent vertigo of unknown cause	Extensive neurotological, including auditory and vestibular function testing and appropriate imaging studies	ICHD3 criteria	Lee et al., 2002 [32]
**Demyelinating disorders**					
Multiple Sclerosis (MS) and Neuromyelitis Optica Spectrum Disorders (NMOSD)	The prevalence of MS in Europe is about 100–190/100.000 inhabitants; the prevalence range of NMOSD is ~ 0.5–4/100.000 worldwide	Isolated vertigo with or without nystagmus	Extensive neurotological, including auditory and vestibular function testing and MRI	Clinical exam, Brain MRI, HIT	Pula et al., 2013 [35], Kremer et al., 2014 [36]
**Infectious**					
Neurocisticercosis	rare	Positional vertigo nystagmus	Cultural tests	Clinical exam, Brain MRI	Joshi et al., 2020 [22]
Cryptococcosis	rare	Fever, vertigo	Cultural tests	Clinical exama, Laboratory tests (CSF culture) neuroimaging (CT, MRI)	Adzic-Vukicevic et al., 2019[34]
**Others**					
Vestibular neuritis	Unknown	Acute onset of vertigo with repetitive falls without hearing loss or tinnitus	recent viral infection	Serology for herpes virus	Lee JY et al., 2019 [37], Roberts RA et al., 2018 [38]
Arnold-Chiari malformation	Rare	Displacement of the cerebellar tonsils	Neuroradiology	Brain MRI	Unal M et al., 2006 [39]
Episodic ataxia type 2	Rare	Paroxysmal recurrent attacks of vertigo which usually respond to the treatment with potassium channel blockers and acetazolamide	autosomal dominant	Genetics	Spacey S et al., 1993 [44]
Hemiplegic migraine	Rare	Acute attack with isolated vertigo or more often associated with hemiparesis and confusion		Clinical exam, genetic testing	Rispoli et al., 2019 [40]
Bowhunter’s syndrome and	Very rare	Recurrent attacks of vertigo associated with neck rotation	Neuroradiology	Dynamic MRI and neurosonology	Di Stefano et al., 2020 [41]
Subclavian steal syndrome	Rare	Recurrent attacks of vertigo associated with the use of an arm	Neuroradiology	MRI and neurosonology	Potter et al., 2014 [42]
Cerebellar syndrome due to naturopathic over-the-counter supplements	Only a single report	Vertigo, gait unsteadiness, nystagmus, hypermetric saccades, dysmetria, ataxia	Anamnesis of supplement use	Clinical exam, Laboratory tests, Neuroimaging	Kim DD et al., 2019 [33]
Frontal lobe epilepsy	Rare	Seizures with onset from the frontal lobe	Antiepileptics (i.e., sodium valproate, levetiracetam, and lamotrigine)	EEG	Jiang et al., 2020 [43]

The most common cause of isolated vertigo in pediatric population is VM, followed by benign paroxysmal vertigo in childhood (BPVC) [7, 14]. Neurovascular diseases, tumors and demyelinating diseases can rarely provoke an altered perception of position in the environment in childhood [7, 16]. More frequent causes of vertigo are orthostatic hypotension, vestibular neuritis (VN) and vestibular paroxysmia [7, 20]. Lastly, a miscellany of conditions causing isolated vertigo are described in children, such as head trauma, drugs, genetic diseases, visual diseases and mental disorders [1, 7, 15, 18, 21, 45].

Conversely, in adulthood, isolated vertigo usually represents the first symptom of an acute vascular disease or a brain tumor [22, 25, 27, 31]. Other possible etiologies are demyelinating disorders, VM and VN [32, 36, 37, 46, 47]. Lastly, isolated vertigo in adults can be associated with genetic disorders, malformations, vascular diseases or psychological illness [22, 33, 34, 39, 42, 48, 49].

## Discussion

Our systematic review revealed that vertigo is a common symptom and an indicator of several diseases in childhood and adulthood. When associated with other symptoms, it is easy to distinguish among differentials. Conversely, when the patient shows isolated vertigo, the diagnostic approach becomes more complex and only through an accurate anamnesis, a complete physical examination and specific tests the clinician can achieve the correct diagnosis. Causes of vertigo in childhood present an age-dependent distribution which may be helpful in narrowing the differential diagnosis. For example, children under five years of age are usually diagnosed with BPVC (71%), followed by VM (19%). These two entities represent the most common causes of vertigo in children under 10 years of age as well, even if with different prevalence (each representing approximately 30% of cases). On the contrary, adolescents are most commonly diagnosed with VM [50]. Also in adulthood causes of vertigo are differently distributed according to age. For example, older patients have a higher burden of vertigo due to cerebrovascular diseases [51]. On the contrary, younger patients more commonly suffer from vertigo due to VM or multiple sclerosis (MS) [35, 52]. The aim of this review is to provide an illustrative overview of isolated neurological vertigo and to design a useful tool for the differential diagnosis of vertigo in the ED.

It needs to be highlighted that bias can arise at all stages of the review process. Hence, we have made more than an effort to minimize the risk of bias in this review identifying possible concerns with the review process. We tried to follow the ROBIS tool for the assessment of bias in the systematic review. Study eligible criteria have been pre-defined to find relevant literature on vertigo to develop an algorithm. The selection of a single database and English language might have limited the search strategy and this may represent a possible source of bias. However, the search strategy was carried out by two authors (NP and VD) independently, with the second reviewer checking the decisions of the first reviewer. Bias may also have arisen from the process of data extraction which is, by its nature, subjective and open to interpretation. Hence, extraction of data was performed by all the author involved working together to develop the algorithm. We calculated an overall level risk of bias as “low-moderate”, according to the ROBIS tool [53].

### Isolated neurological vertigo in childhood

Vertigo in pediatric age is a challenge due to different etiopathologies, the short-lived manifestations owing to rapid compensation, the inability of children to describe the experienced symptoms and the feasibility of diagnostic tests [5].

In childhood, the most common causes of isolated neurological vertigo are VM and BPVC, although frequencies vary between different studies [6, 12, 19]. In opposition to adulthood, cerebral vascular diseases, brain tumors and demyelinating disorders are uncommon causes [4, 6, 7, 54]. Differential diagnosis include also orthostatic hypotension, VN and vestibular paroxysmia [1, 20, 55]. Finally genetic diseases, head trauma, drugs, visual and psychiatric disorders should be taken into account while evaluating a child with isolated vertigo [1, 7, 15, 18, 21, 45].

In this systematic review we proposed an algorithm to facilitate the approach to children presenting to the ED with isolated vertigo, based primarily on a detailed history and a careful examination.

### Vestibular migraine

VM is the most common cause of vertigo in children, accounting for 24% of all vertigo causes and is more frequently observed in adolescents [6]. It is characterized by a range of signs and vestibular manifestations temporally associated with migraine [56]. However, presentation may be variable and the onset of vertigo can precede the development of the headache by many years [57].

Patients typically present with episodic attacks of spontaneous or positional vertigo that can last between 5 min and 72 h and can be associated with headache, photophobia and phonophobia [6, 57]. The combination of vestibular manifestations and headache is commonly observed, but less than 50% have both symptoms in every attack. During the acute attack, patients can develop pathological nystagmus, meanwhile between the attacks the neurological examination is usually normal. In contrast to headache, vertigo does not respond well to triptans or non-steroid analgesics [58]. Finally there are no specific biomarkers and the diagnosis relies mainly on the patient medical history [57].

### Benign paroxysmal vertigo in childhood

BPVC is an episodic syndrome with short (seconds to minutes) recurrent attacks of subjective or objective vertigo, which resolves spontaneously. BPVC is a common cause of vertigo in childhood and it precedes the onset of migraine in over 35% of children by many years [4, 7, 59]. A family history of migraine is present in half of patients [59]. Children under the age of five and females are mainly affected. BPVC needs to be distinguished from Panayiotopoulos syndrome, a benign epileptic syndrome that can present also with vertigo and it is characterized by repetitive nature and association with autonomic symptoms [45]. Clinical exam and instrumental investigations are normal with no hearing impairment [14, 59].

### Neurovascular diseases

Arteriovenous malformations presenting with vertigo symptoms are more frequent in the pediatric population (35%) than in adults (6%) and symptoms are related to the compression of the vestibular nerve or the brainstem nuclei [4]. Among neurovascular disorders, posterior circulation stroke represents approximately 3% of children with vertigo, usually secondary to cervico-cerebral artery dissection [60]. Other vascular diseases responsible for central vertigo are represented by cerebellar infarction, hemorrhage, occlusion of the basilar artery and dissection of the vertebral artery. Among these causes, Wallenberg's syndrome, also known as lateral medullary infarction, causes vertigo [17, 54]. Moreover vasculitis associated with rheumatologic disorders such as Wegener’s granulomatosis, systemic lupus erythematosus and juvenile rheumatoid arthritis could cause vertigo related to impaired vertebrobasilar circulation [61]. Indeed Moya-Moya disease, characterized by stenosis of the intracranial carotid arteries and basal collateral arteries, can be associated with vertigo on standing [62]. In these cases, a full physical examination usually helps to find out other associated neurologic deficits.

### Tumors

Brain tumors are not a common cause of vertigo in children. Tumors of the posterior cranial fossa rarely begin with vertigo in the pediatric population (< 1%). More commonly they present with headache, vestibular symptoms or additional neurological deficits due to the compression or involvement of the nearby nuclei and fiber tracts [6, 7, 63]. Although rare, medulloblastoma and other cerebellar tumors have been reported to cause vertigo in children [6, 7].

### Demyelinating diseases

Vertigo, usually lasting days or weeks, has been reported as initial sign of MS in 20–50% of patients [54]. The acute manifestation of vertigo in MS falls into two categories: acute vestibular syndrome as central form and BPPV as peripheral form [35]. It is then necessary an adequate differential diagnosis, that can be difficult in MS with atypical central signs [13, 35]. Cochleo-vestibular dysfunction with vertigo have been described also in chronic-inflammatory-demyelinating neuropathy [64].

### Orthostatic hypotension

Orthostatic hypotension determines vertigo in 3–9% of symptomatic children. Affected children become symptomatic within 3 min of moving from posture to sitting or standing from a supine position. It can occur as isolated symptom or associated with autonomic dysfunctions [6]. It is fundamental to exclude life-threatening cardiac origin and to assess blood pressure also to rule out hypertension, which can in turn cause vertigo [7].

### Vestibular neuritis

VN represents between 1 and 16% of cases of pediatric vertigo and it is commonly founded in children older than five years of age and adolescents [65]. It is an acute inflammation of the vestibular component of the eighth cranial nerve that primarily occurs after an acute upper-respiratory viral infection, mainly due to herpes simplex, but also to Adenoviruses and Enteroviruses [6]. Affected children present with a sudden onset of severe vertigo without hearing loss that lasts for few days or weeks and resolves in a one-month period. It can be often intensified by small changes in head position and can be associated with nausea and vomiting [65]. On examination the clinician can elicit a horizontal-rotary spontaneous nystagmus with quick phases to the unaffected side but the neurological exam can be completely normal at the time of presentation. Diagnosis is made by electronystagmography and thermal caloric testing [65].

### Vestibular paroxysmia

Though rare, vestibular paroxysmia can cause multiple short episodes of rotatory or to-and-fro vertigo, up to 30 times or more in a day, that last from seconds to minutes [20]. In some patients, attacks are unprovoked but sometimes head movements or hyperventilation can elicit them [66]. Imaging may demonstrate the neurovascular compression of the vestibulocochlear nerve at the root entry zone and to rule out brain tumors [6, 66]. Distinctive findings include hyperventilation-induced nystagmus and mild vestibular impairment on caloric testing, as well as the good response to carbamazepine [6, 20].

### Genetic diseases

Familial episodic ataxia type II is a rare autosomal dominant disorder characterized by vertigo that lasts for minutes to hours and ataxia, typically triggered by sport, stress and alcohol [21]. This disease is due to a mutation in the CACNA1A-gene, coding for a subunit of the P/Q calcium channel, and it has its onset in childhood or adolescence. Carbonic anhydrase inhibitor produces a complete response to vertigo [21]. Similar episodes have been reported in patients affected by hemiplegic migraine, especially from ATP1A2 mutations [40, 41, 67].

Tuberous sclerosis is an autosomal dominant disorder characterized by hamartic development in several organs, most notably brain, heart, kidneys, lungs and skin. To date, only one case report described a 9-year-old patient with episodes of vertigo and headache followed by full spontaneous recovery as initial symptom of tuberous sclerosis [15].

### Other causes of vertigo

Head trauma from falls and whiplash injury are possible cause of vertigo among children, owing to the labyrinthine concussion or the development of perilymphatic fistula [6, 7]. Children affected by post-traumatic vertigo tend to exhibit abnormal results to vestibular tests in nearly half of the cases. Neuroimaging is mandatory to detect possible fractures or brain lesions [2, 6].

Despite a large number of drugs including vertigo as a possible side effect, iatrogenic forms are rarely reported in children [1].

Anisometropia [6] and other binocular vision disorders, including vergence abnormality [18] are other possible causes of vertigo due to sensory mismatch; ophthalmological evaluation and treatment are mandatory.

Finally, the differential diagnosis of childhood vertigo comprises somatoform vertigo, commonly found in adolescent girls that usually present with isolated episodic or chronic vertigo with normal findings on physical examination [14]. A careful clinical work-up to rule out potential diseases and a psychiatric consultation are essential [6].

### Isolated neurological vertigo in adulthood

In adulthood, vertigo can occur as a result of conditions related to impaired cerebral circulation, especially of the vertebrobasilar district [23] or it can be the first sign of intra-axial and extra-axial brain tumors, particularly of primary tumors of the posterior cranial fossa and cerebellar metastases [22, 68]. Isolated vertigo is also associated with migraine [56] and neuro-immunological diseases [47]. Lastly, there are rare genetic diseases [44], malformation syndromes [39] and vascular disorders [42, 69] whose clinical manifestation, at the onset, can be represented by vertigo. To date, there are few data on functional pathological vertigo, which remains a diagnosis of exclusion [49].

As per children, we proposed a diagnostic algorithm to guide clinician’s approach to adults with isolated neurological vertigo in ED.

### Ischemic stroke

Acute isolated vertigo can frequently occur in patients suffering from stroke in the distribution of the vertebrobasilar circulation [23]. The frequency of isolated vertigo in stroke ranges from 11 to 29% but it is probably underestimated [28].

Differentiating isolated vascular vertigo from other disorders involving the inner ear is difficult, especially when AICA territory, that supplies both peripheral and central vestibular structures, is involved [29]. Patients can develop typical BPPV as a consequence of labyrinthine ischemia [23], which cannot be detected with current imaging techniques [70]. The most salient feature which distinguishes central positional vertigo (CPV) from BPPV is atypical direction of nystagmus for the stimulated canal during repositioning maneuvers [71] that usually shows an initial peak and a subsequent decrescendo pattern [72].

Vertigo can be the only presenting symptom in patients with posterior inferior cerebellar artery (PICA) infarction [26] and it is considered rare in superior cerebellar artery (SCA) infarction, which does not have significant vestibular connections [73].

Isolated vertigo can result from dorsal medullary infarction, which can involve vestibular nuclei, nucleus prepositus hypoglossi or inferior cerebellar peduncle [30]. Clinical signs of acute peripheral vestibulopathy have been reported in patients with pontine lesions, as a consequence of vertebrobasilar ischemia [74].

Isolated intermittent vertigo can be rarely the consequence of hypoperfusion of the flocculo-nodular lobe due to a persistent trigeminal artery (PTA) or posterior circulation stroke in the context of a PTA [31]. Finally, an isolated vestibular-like syndrome (VLS) has been recently reported in patients with ischemic strokes confined to the insula [75].

Almost all the patients with vascular vertigo show central vestibular signs, such as gaze-evoked nystagmus, normal or contralesional head-impulse test (HIT), skew deviation and central patterns of spontaneous nystagmus [30]. Head-Impulse, Nystagmus, Test of Skew (HINTS) is a three-step examination method which has 100% sensitivity and 96% specificity for stroke [11].

### Tumors

Brain tumors account for approximately 2% of all cancers [76]. Even if most patients manifest with a large variety of neurological symptoms, few may present just with vertigo at the onset [22], particularly tumors of posterior fossa [77]. However, an accurate examination may often show additional nystagmus with central features (see above). The most frequent neoplastic diseases at this site are cerebellar metastases (intra-axial) and vestibular schwannoma (extra-axial). Primary intra-axial posterior fossa tumors may involve cerebellum, brainstem or the fourth ventricle [68]. Among extra-axial ones a meningioma may also cause isolated vertigo [78]. Furthermore, it is worth to mention a non-neoplastic mass effect lesion which may frequently cause vertigo constituted by arachnoidal cyst sited in the posterior fossa [79].

### Migraine

Migraine should always be considered among the causes of isolated neurological vertigo because vertigo may be one of the symptoms preceding or manifesting during migraine attack [80]. Furthermore, in the last International Classification of Headache Disorders there is even a definite condition called “vestibular migraine” that may reveal itself as intermittent episodes of vertigo [56].

### Multiple sclerosis and other demyelinating disorders

Unexpected diagnoses of MS have been made in patients who had an isolated positional vertigo as the only symptom [81]. Responsible lesions are often in the intra-pontine eighth nerve fascicle and oculomotor signs can be associated [35]. In these cases, neuro-otological examination might show a normal HIT with suppression of transient evoked otoacoustic emission [81]. Brain MRI, as well as oligoclonal IgG bands in cerebrospinal fluid (CSF) should be recommended, especially in young women with isolated positional vertigo and a normal HIT [35].

Even neuromyelitis optica spectrum disorders (NMOSD) can rarely present with isolated vertigo, especially early in the disease course [36]. Vertigo and nystagmus can precede typical manifestations of neuritis optica and myelitis, as a consequence of lesions in the medulla, cerebellum or pons [46]. Therefore search for antiacquaporin-4 antibodies and anti-myelin oligodendrocyte antibodies should be taken into consideration in selected cases [35]

### Vestibular neuritis

VN is a condition caused by inflammation of the vestibular nerve commonly seen in middle-aged adults [47] as consequence of a viral infection (frequently related to herpes zoster virus). In VN, there is an acute onset of isolated vertigo without hearing loss or tinnitus but sometimes patients present with repetitive falls. Vertigo is severe, lasting for 2–3 days and is usually followed by gradual recovery in few weeks; symptoms and diagnostic test can change in relation to nerve’s involvement site [37, 82]. This condition should be suspected in patients who presented a recent viral infection, especially when taking immunosuppressive drugs [38].

### Rare causes

Arnold-Chiari malformation is a rare condition in which a displacement of the cerebellar tonsils can affect functions controlled by the cerebellum and brainstem thus causing vertigo[39].

Episodic ataxia type 2 is a rare condition, allelic with hemiplegic migraine and spinocerebellar ataxia, caused by an autosomal dominant mutation in the CACNA1A gene resulting in the dysfunction of voltage-dependent calcium channels [41]. The onset is in fifth-seventh decade and patients suffer from paroxysmal recurrent attacks of vertigo which usually respond to potassium channel blockers and acetazolamide [44].

Repetitive vascular compressions in vertebral arteries (bowhunter’s syndrome) [69] or atherosclerosis in subclavian artery (subclavian steal syndrome) [42] are both conditions in which recurrent attacks of vertigo occur owing to an impaired vascular flow in posterior circulation [69]. However, even if vertigo can be the only reported complaint, there are usually associated neurological signs such as nystagmus, gaze palsy, pupillary defects or sensory-motor deficits [69]. Rarely, in adult age, vertigo has been reported in frontal lobe epilepsy as the sole ictal symptom at the seizure onset, or, more frequently, followed by other ictal signs/symptoms [43]. Finally, functional isolated vertigo has been rarely reported [49].

## Conclusions

Our systematic review demonstrates that isolated neurological vertigo could be due to different causes in childhood and adulthood. VM and BPVC are the most frequent disorders in children suffering from isolated vertigo presenting the ED; meanwhile the same symptom in adults is more frequently related to impaired distribution of the vertebrobasilar circulation, especially in the older ages. Age may be helpful in narrowing the differential diagnosis. In the majority of the cases, an appropriate diagnosis can be established thorough a careful history collection and a complete clinical examination, as illustrated in the algorithms proposed in the paper that could be an important tool for a prompt differential diagnosis. Indeed it is crucial to be aware of those differentials for pediatric and adult age to be able to make the proper diagnosis and manage those patients appropriately.

## Data Availability

All data used and/or analysed during this study are included in this published article.

## Abbreviations

ED: Emergency Department
VM: Vestibular migraine
BPVC: Benign paroxysmal vertigo in childhood
VN: Vestibular neuritis
MS: Multiple sclerosis
BPPV: Benign paroxysmal positional vertigo
AICA: Anterior inferior cerebellar artery
CPV: Central positional vertigo
PICA: Posterior inferior cerebellar artery
SCA: Superior cerebellar artery
PTA: Persistent trigeminal artery
VLS: Vestibular-like syndrome
HIT: Head-impulse test
HINTS: Head-Impulse Nystagmus Test of Skew
NMOSD: Neuromyelitis optica spectrum disorders
